# Antiviral Activity of Metal-Containing Polymers—Organotin and Cisplatin-Like Polymers

**DOI:** 10.3390/ma4060991

**Published:** 2011-05-27

**Authors:** Michael R. Roner, Charles E. Carraher, Kimberly Shahi, Girish Barot

**Affiliations:** 1 Department of Biology, University of Texas Arlington, Arlington, TX 76019, USA; 2 Department of Chemistry and Biochemistry, Florida Atlantic University, Boca Raton, FL 33431, USA; E-Mail: carraher@fau.edu; 3 Florida Center for Environmental Studies, Palm Beach Gardens, FL 33410, USA; 4 Department of Biological Sciences, University of North Texas, Denton, TX 76203, USA; E-Mail: kimberly.shahi@unt.edu; 5 Department of Biology, Boston University, Boston, MA 02215, USA; E-Mail: irishbarod@yahoo.com

**Keywords:** organotins, antivirals, antitumor activity, polymers, dibutyltin, kinetin, diethylstilbestrol, dienestrol, platinum-containing polymers, cisplatin, tilorone, methotrexate

## Abstract

Polymers containing platinum and to a lesser extent tin, have repeatedly demonstrated antitumor activity *in vitro* and *in vivo* against a variety of cell and tumor types. The mechanisms responsible for the antitumor activity include inducing a delay in cell proliferation and sister chromatid exchanges blocking tumor growth. As most DNA and some RNA viruses require, and even induce, infected cells to initiate DNA replication and subsequent cell division, compounds with antitumor activity will very likely also possess antiviral activity. This article examines the use of metal-containing polymers as a novel class of antivirals.


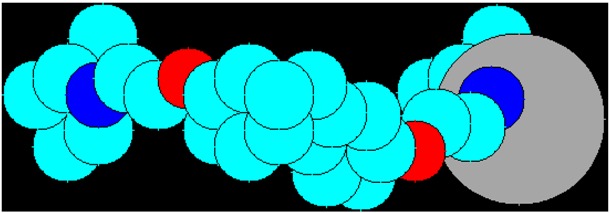


Repeat unit of the product of potassium tetrachloroplatinate II and tilorone

## 1. Introduction

This paper brings together results from a number of studies of ours over the past few years describing results from investigating the antiviral activity of organotin and cisplatin-like polymers that represent two new groups of compounds that offer antiviral activity. While the studies demonstrate that some of these products exhibit good antiviral activities, they represent just a beginning and much needs to be done before coherent trends are known.

It is not surprising that metal-containing moieties exhibit an effect on many of the essential units within our biosphere. Most metals and metal-containing units possess vacate p, d, and f orbitals that can interact with a variety of biologically entities. The site(s), extent and biological result of these interactions varies widely.

A number of classic chemotherapeutic compounds have demonstrated not only antitumor but unexpected antiviral activities [1,2]. The hypothesis is that cells infected with most DNA viruses and some RNA viruses normally demonstrate increased cellular DNA replication, a condition also seen in transformed cells. A wide variety of metal-containing compounds, including those contained within a polymer, are known to offer antitumor activity. These include gold, ruthenium, vanadanium, copper, titanium, iron (ferrocene), lanthanides and various metal ions. Here our focus will be on organotin and platinum-containing compounds. Organotins are known to have broad antitumor activity [3,4,5,6,7,8,9,10,11,12,13,14,15,16,17,18,19,20,21,22,23,24,25,26,27,28,29,30,31,32,33,34,35,36,37,38,39,40,41,42,43,44,45,46,47,48,49,50,51,52,53,54] through different mechanisms including the inhibition of cellular DNA replication [7,55,56]. Cisplatin and related compounds are some of the most widely used anticancer drugs. The main site of activity is believed to be on the DNA itself. The topic of platinum-containing compounds including polymers has been reviewed including their anticancer activity [57,58,59,60,61,62]. Here we describe some of results related to their ability to inhibit virus growth.

## 2. Results and Discussion

### 2.1. Antiviral Activity of Organotin Polymers

Surprisingly little work has been done regarding the use of organotin compounds as antiviral agents. Two studies examined the ability of monomeric organotin compounds to act as antiviral agents. Octahedral organotin complexes of the form R_2_SnX_2_L_2_ where R = Et or Ph, X = Cl or Br, and L_2_ = o-phenanthroline or 2-(2-pyridyl)benzimidazole, have shown *in vitro* antiherpes activity toward both herpes simplex virus types 1 and 2 (HSV-1 and HSV-2) [63]. In addition, a series of mono, di, and tri-organotin halided (alkyl and phenyl) exhibited weak antiherpes activity in the same viral assay system.

In a related study [64], this group looked at the same group of compounds against both DNA (herpes simples virus types 1 and 2), a TK-(thymidine kinase deficient) strain of herpes simplex virus type 1, and vaccinia virus. The RNA viruses were vesicular stomatitis virus, coxsackie virus type B4, sindbis virus type 3, and human immunodeficiency virus (HIV). Overall, the complexes exhibited weak antiviral activity and low selectivity. Most of the complexes were active against one or more of the three strains of herpes simplex viruses. By comparison, only three complexes were active against some of the RNA viruses. None of the compounds were active against vesicular stomatis or parainfluenza virus or HIV virus.

We have found that a wide variety of tin-containing polymers exhibit antiviral activity [32,34,45,60,61,65,66].

In investigating the correlation between structure and ability to inhibit cancer cell lines, we studied a variety of organotin polymers including a large group of organotin polyethers. Much of the effort with these organotin polyethers focused on maintaining the same organotin and varying the hydroxyl-containing moiety. The dibutyltin moiety, derived from dibutyltin dichloride, was chosen as the organotin unit for several reasons. First, it is the least expensive of the organotin halides and available in the ton and greater quantity. Second, in the vast majority of studies by us and others where the identity of the organotin was changed, monomeric and polymeric compounds containing it exhibited the best ability to inhibit cancer cell lines. Third, it is the least toxic of the lower alkyltins to humans. Fourth, commercially it is the most widely employed organotin moiety.

The products described here are all polymeric with degrees of polymerization greater than fifty. Further, these polymers exhibited a good ability to inhibit a variety of cancer cell lines, including those associated with ovarian, colon, lung, prostate and breast cancers, at concentrations equal to and less than the control, cisplatin, the most widely employed anticancer drug.

The moiety attached to the organotin is critically important to its antiviral activity, and the activity is strongly virus-specific, even among viruses with similar genome types. This is demonstrated in the following descriptions.

**Figure 1:**
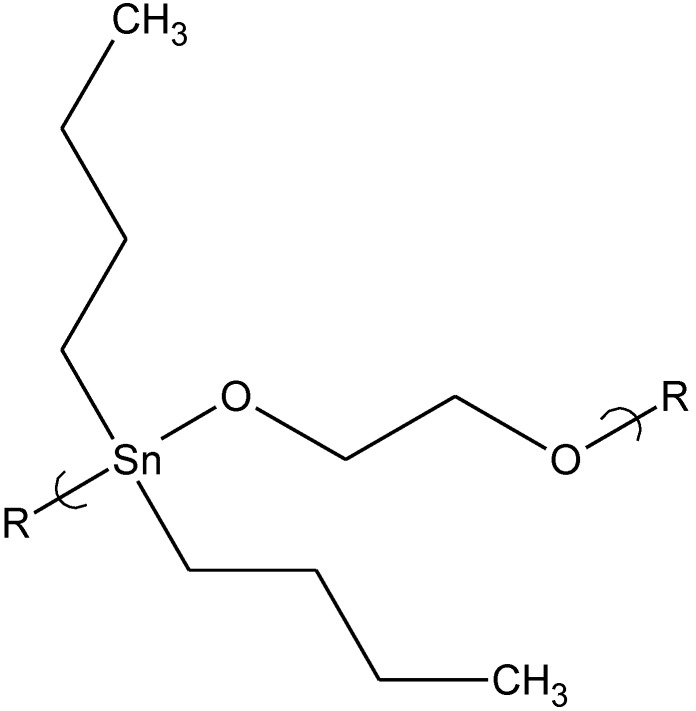
Repeat unit for the product from ethylene glycol and dibutyltin dichloride (In all cases, the R simply represents chain extension).

The initial series of organotin polyethers studied employed a variety of aliphatic diols including ethylene oxide-like products (Figure 1) [11,12,31,36,49,51].

**Figure 2:**
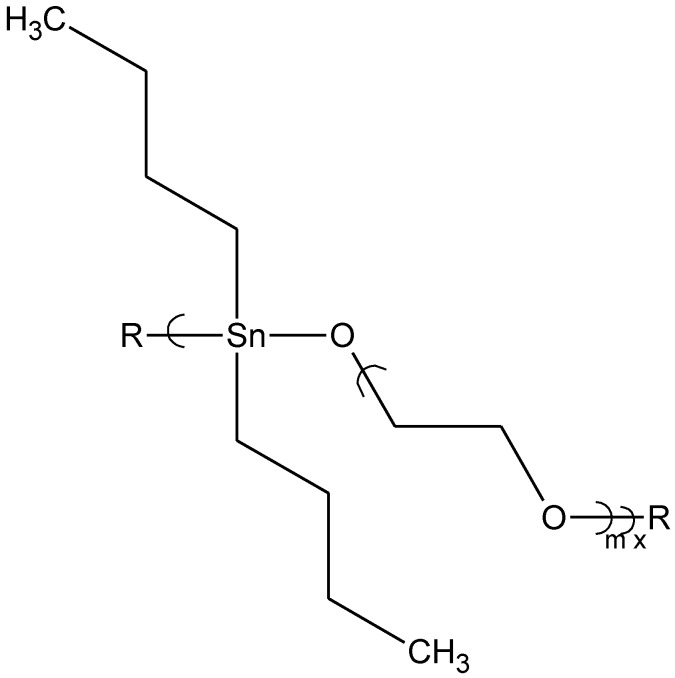
Repeat unit for the product of poly(ethylene glycol) and dibutyltin dichloride.

While many exhibited some antiviral activity, only a few exhibited significant antiviral activity. One was derived from the simple diol ethylene glycol (Figure 1). Another was derived from hydroxyl-capped polyethylene glycol, PEG (Figure 2). The PEG product deserves special comment. It is the first water-soluble organotin polymer and as such would allow simple delivery of the compound using any of the typical methods of delivery. Most of the products are not water soluble so DMSO samples are made and water is added to secure the appropriate test samples. Generally, the amount of DMSO is much less than 1% by volume. This form of delivery though initial solubilizing of the test material in DMSO is the most widely employed industrial approach to effect compound solubility.

Studies were carried out using the HSV-1 and vaccinia viruses. Both of these viruses have a genome of double-stranded DNA. HSV-1 replicates in the nucleus of infected cells while the vaccinia virus, a member of the poxvirus family, replicates in the cytoplasm of infected cells. The product from dibutyltin dichloride and ethylene glycol (Figure 1) had high activity against the vaccinia virus, but not against the HSV-1 virus. Of the polyethers studied, the ethylene glycol product has the shortest distance between the dibutyltin moieties within the polymer chain making it the most polar and most susceptible to degradation. It is possible that the active compound moiety is derived from the degradation products making it easier for these small units to enter the cell. This would account for why it could inhibit vaccinia viral production, but not HSV-1 viral production. 

By comparison, the PEG polymer (Figure 2) is water soluble and this water solubility could account for the good activity against both the vaccinia and HSV-1 viruses allowing it ready access to both the cell and cytoplasm.

**Figure 3:**
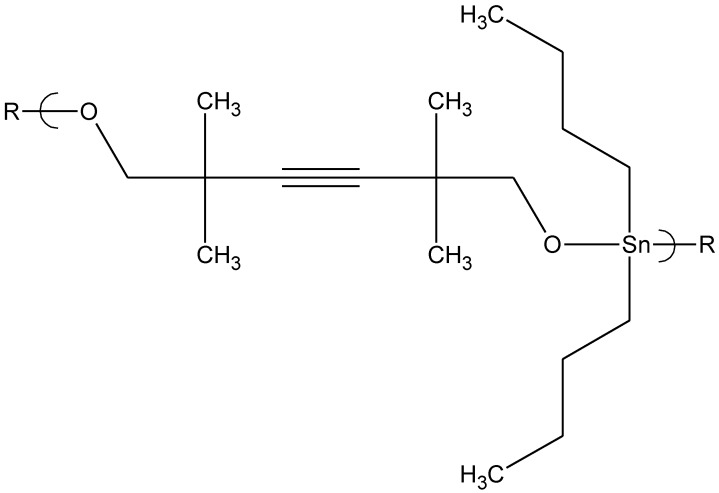
Repeat unit for the product of dibutyltin dichloride and 2,5-dimethyl-3-hexyn-2,5-diol.

Dibutyltin chloride (Bu2SnCl2) itself has similar antiviral activity against the two viruses, herpes simplex type-1 (HSV-1) and vaccinia virus. Bu_2_SnCl_2_ at a concentration of 0.15 μg/mL is not cytotoxic but blocks virus replication in approximately 20% of virus exposed cells. Cells treated with a water-soluble polymer derived from PEG (400 amu) and dibutyltin dichloride demonstrate an increased protection from infection by vaccinia virus, where greater than 30% of the treated cells are protected from virus infection at compound concentrations 10-fold lower (0.015 μg/mL) than the dibutyltin dichloride alone. At a concentration of 0.15 μg/mL, the polymer derived from 2,5-dimethyl-3-hexyn-2,5-diol and dibutyltin dichloride (Figure 3) protects almost 50% of the treated cells from infection by HSV-1 [12]. These examples of organotin polyether polymeric bioactive materials demonstrate that the moiety attached to the organotin is critically important to its antiviral activity, and that the activity is strongly virus-specific, even among viruses with similar genome types.

**Figure 4:**
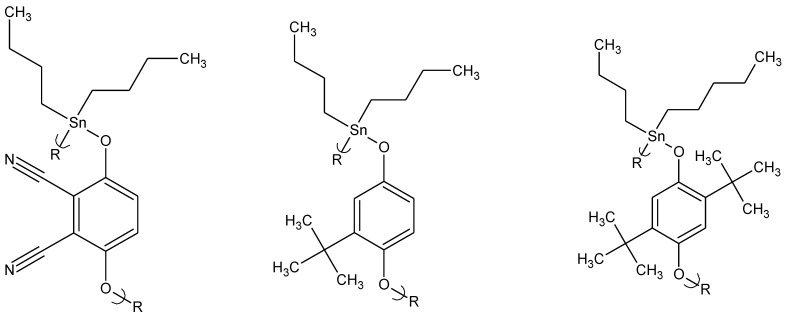
Repeat units for the dibutyltin polyethers derived from (left to right) 2,3-cyanohydroquinone; t-butylhydroquinone; and 2,5-di-t-butylhydroquinone.

Along with the organotin polyethers derived from aliphatic diols, we also synthesized polymers derived from aromatic diols including hydroquinone and hydroquinone derivatives (Figure 4) [32]. As a group, these polymeric drugs demonstrated the highest antiviral activity of the organotin polyether drugs tested. The highest activity against HSV-1 was seen from 2,3-dicyanohydroquinone (Figure 4, left); tert-butylhydroquinone (Figure 4, middle) and 2,5-ditertbutylhydroquinone (Figure 4, right). All three polymers are active at a concentration of 0.30 μg/mL, and block HSV-1 infection in 35% (2,3-dicyanohydroquinone polymer) to almost 80% (2,5-ditertbutylhydroquinone polymer) of the polymer treated cells. Only the tert-butylhydroquinone polymer is active against vaccinia virus, protecting more than 25% of the treated cells from virus infection. The aromatic hydroquinone ring structure of these polymeric bioactive materials may interact through pi-related secondary bonding with the various entities within the cell including the pyrimidine and purine rings on the DNA allowing them their activity within the cell. The combination of the active sites on the tin and these pi-interactions may be the reason behind the high activity of these polymeric drugs. The polymeric nature of the bioactive materials may also interact with the virus outside of the cell increasing the difficulty of the virus attaching to the cell. As in all of these cases, much more needs to be done to actually determine how and where vital activity occurs.

It is of interest to note that the polymer derived from 2,5-dimethyl-3-hexyn-2,5-diol also contains pi bonding and it also exhibits antiviral activity as noted before. 

A third group of polymers, organotin polyamines, derived from reaction of various 4,6-diaminopyrimidines and dibutyltin dichloride was studied. Upon evaluation a number of these polyamines compounds including those derived from 4,6-diaminopyrimidine; 4,6-diamino-5-nitropyrimidine; 4,6-diamino-2-methylmercaptopyrimidine; 4,6-diamino-2-methyl-5-nitrosopyrimidine; 4,6-diamino-2-mercaptopyridine; 4-chloro-2,6-diaminopyrimidine; 2,4-diamino-6-hydroxypyrimidine; 4-diamino-6-hydroxy-5-nitrosopyrimidine; and pyrimethamine exhibited some ability to inhibit viral growth. The antiviral activity is most likely due to 4,6-diaminopyrimidine-derived products being a nucleoside analog. The virus may incorporate the polymer or units derived from it, into its nucleic acid instead of the usual pyrimidine.

**Figure 5:**
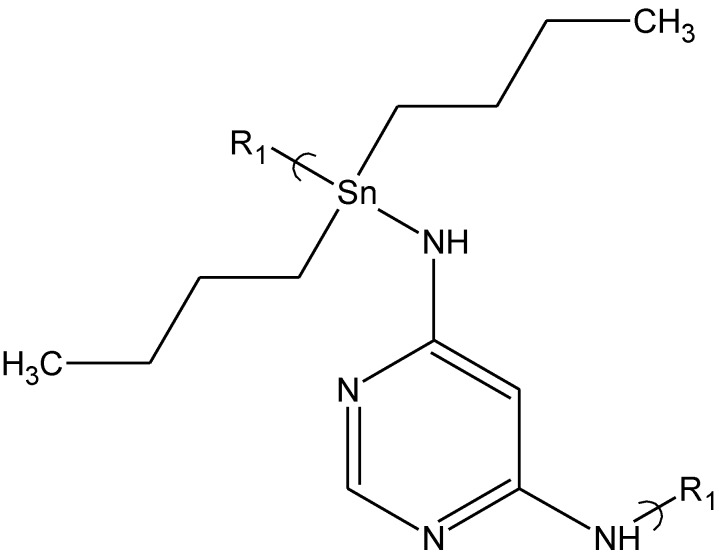
Repeat unit for the product of dibutyltin dichloride and 4,6-diaminopyrimidine.

Even so, only a few exhibited significant viral activity. The polyamine derived from 4,6-diaminopyrimidine (Figure 5) at a concentration of 0.30 μg/mL blocks virus replication in approximately 30% of HSV-1 virus exposed cells but is unable to prevent the vaccinia virus infection in similarly treated cells. In contrast, treatment of cells with the 4,6-diamino-2-mercaptopyridine product protected approximately 20% of the cells from infection by either HSV-1 or vaccinia virus. Again, specific activity is dependent on the nature of the Lewis base.

**Figure 6:**
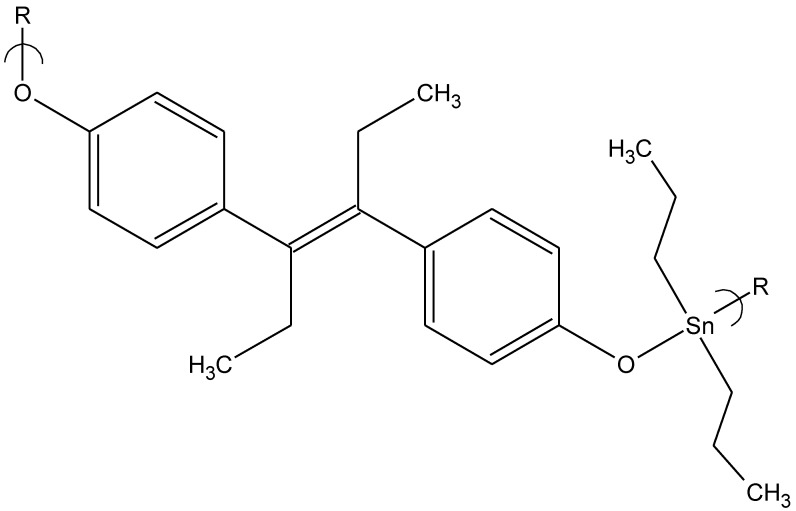
Repeat unit for the product of dipropyltin dichloride and diethylstilbestrol.

Another group of organotin polyethers was studied employing different organotin moieties (dimethyltin dichloride, diethyltin dichloride, dipropyltin dichloride, dibutyltin dichloride, dicyclohexyltin dichloride and diphenyltin dichloride) reacted with diethylstilbestrol [14]. Again, while several exhibited some activity the dipropyltin product (Figure 6) showed strong antiviral activity against vaccinia virus, preventing more than 30% of treated cells from infection at a concentration of 0.15 μg/mL. This indicates that all of the organotin moieties should be studied with respect to antiviral activity and not only those containing the dibutyltin moiety to gain a full measure of the viral activity of these polymers.

**Figure 7:**
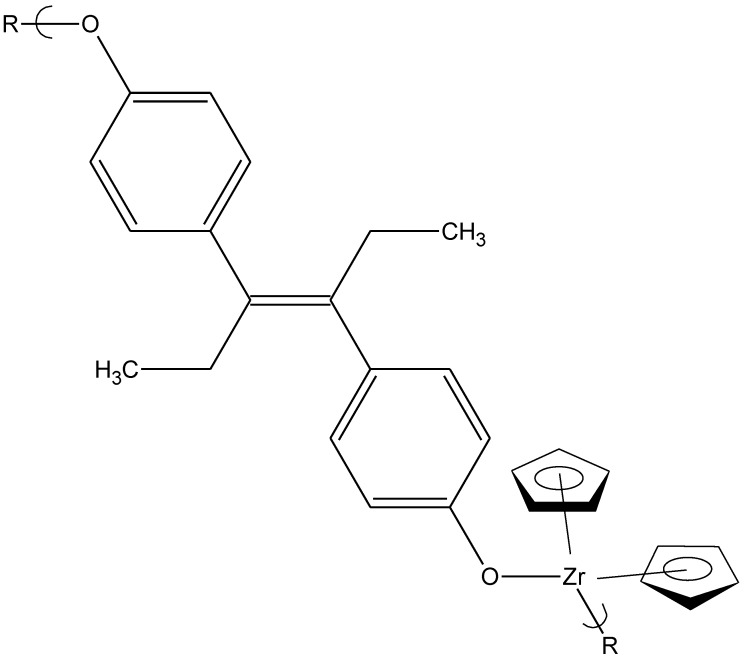
Repeat unit for the product of bis(cyclopentadienyl)zirconium dichloride and diethylstilbestrol.

To demonstrate that other metal-containing products exhibit antiviral activity, the product from bis(cyclopentadienyl)zirconium dichloride and diethylstilbestrol (Figure 7) was also active against vaccinia virus, preventing more than 35% of treated cells from infection at a concentration of 0.15 μg/mL [24].

### 2.2. Acyclovir

**Figure 8:**
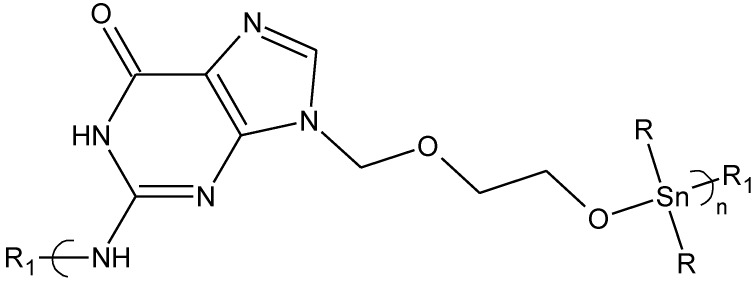
Repeat unit for the product derived from reaction of acyclovir with organotin dihalides.

The inhibitory activity of acyclovir is highly selective [34,60]. Acyclovir is widely used to inhibit several herpes viruses, particularly HSV-1 and HSV-2. It is also used to treat varicella-zoster virus (VZV), Epstein-Barr virus (EBV), and the cytomegalovirus (CMV). Thus, acyclovir is a first line antiviral drug. We synthesized a variety of products focusing our antiviral efforts on organotin materials (Figure 8) [34].

Results of antiviral studies showed several things. First, in all cases many of the polymers out performed acyclovir itself. The performance is even greater when compared with the amount of acyclovir present in each sample. In general, the amount of acyclovir within the polymers represents about one half of the weight of the polymer so that all of the polymers out performed acyclovir itself on a total possible amount of acyclovir moiety present. Second, the order of inhibition, based on the concentration needed to effect 50% inhibition is HSV-1 > VZV > Vaccinia WR > Reovirus ST3 with little inhibition found for the reovirus but outstanding inhibition found for HSV-1 and VZV viruses. Third, the order of viral growth inhibition is similar for each of the viruses and also similar to the order of GI values found for the cancer cell lines. The trend with respect to VZV is the most divergent of the trends but it still has dibutyltin and diethyltin inhibiting at the lowest concentrations. For HSV-1 the order is dibutyltin > diethyltin > diphenyltin = dioctyltin > acyclovir > dicyclohexyltin and for VZV the trend is diethyltin > dibutyltin > dioctyltin > diphenyl > dicyclohexyltin > acyclovir.

The minimum inhibition concentration, MIC, values are much lower indicating that for viral infections where the number of viruses are not great, that the polymers and acyclovir itself should demonstrate good antiviral activity. The overall trend is approximately dibutyltin > diethyltin > diphenyltin > dioctyltin > acyclovir > dicyclohexyltin again similar to that found in other parts of the study that focused on cancer.

These studies are consistent with some of the organotin polymers, namely the dibutyltin, diethyltin and diphenyltin polymers, offering superior inhibition in comparison to acyclovir. Again, these trends are accentuated when considering that only about half of the polymers weight is derived from acyclovir. Thus, the activities are not due to the acyclovir alone, but are enhanced either because of the presence of the organotin moiety, presence of the acyclovir within a polymer, through control release of the acyclovir, or some combination of these factors.

### 2.3. Antiviral Activity of Platinum-Cisplatin Derivatives

**Figure 9:**
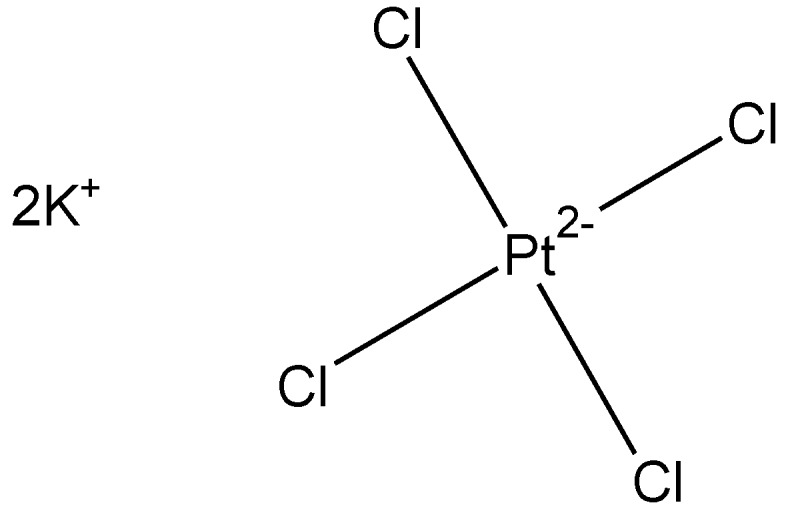
Potassium tetrachloroplatinate II.

To expand on earlier work that suggests platinum-cisplatin derivatives may demonstrate broad antiviral activity, we selected viruses with either RNA or DNA genomes. A number of viruses were studied including the reovirus, herpes simplex virus, varicella virus, and the varicella zoster virus [67,68,69,70,71]. We found that potassium tetrachloroplatinate II itself (Figure 9) showed very limited ability to prevent virus infection of susceptible cells by any of these viruses. The minimum inhibitory concentrations for these viruses were greater than 75 g/mL.

Here we will briefly discuss results of cisplatin-like polymers from four Lewis base units, two tilorones , tetramisole, and methotrexate, all synthesized from the reaction between tetrachloroplatinate and the corresponding Lewis base.

**Figure 10:**
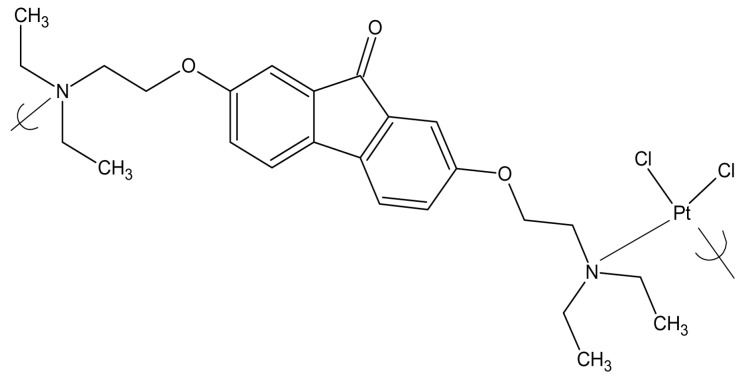
Repeat unit for the product of potassium tetrachloroplatinate II and tilorone.

**Figure 11:**
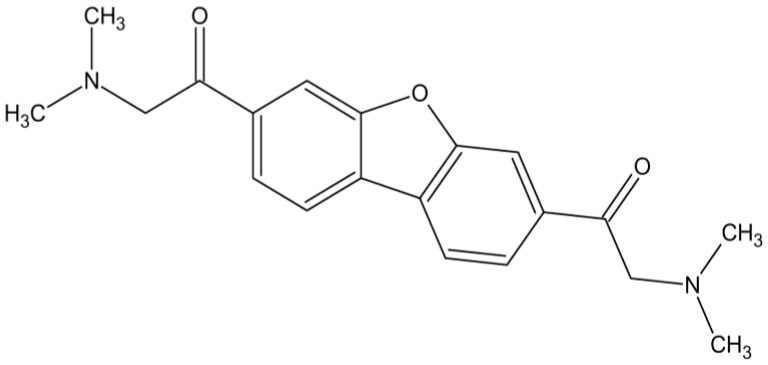
Repeat unit for the product of potassium tetrachloroplatinate II and tilorone 11,567.

Polymers were synthesized from two tilorones [72]. Both of the tilorone polymers (Figure 10 and Figure 11) exhibited good inhibition of all of the tested viruses at low concentrations.

**Figure 12:**
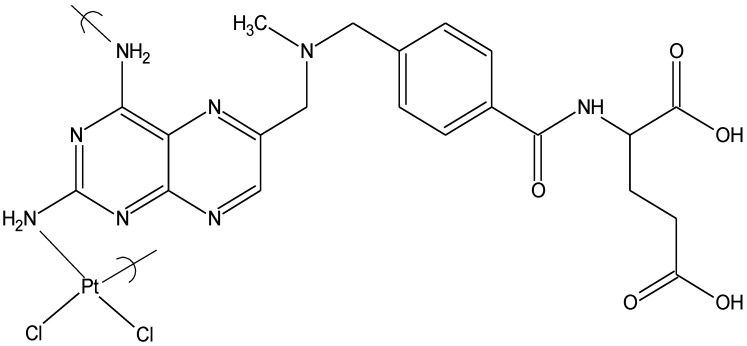
Repeat unit for the product of methotrexate and tetrachloroplatinate II.

In comparison, the organotin polymers of norfloxacin, ampicillin and acyclovir demonstrated inhibition of these three viruses also, but at much higher concentrations, within the range of 2 μg/mL [73,74], while the tilorone polymers showed good activity at 0.1–0.2 μg/mL, well below that of the organotin polymers. The analogous cisplatin polymer derived from methotrexate (Figure 12) showed good activity against HSV-1 (about 4 μg/mL) and VZV (about 6 μg/mL). A limited amount of antiviral activity is seen against vaccinia virus, the DNA virus with cytoplasmic DNA replication and RNA transcription, both processes utilizing viral polymerases. No antiviral activity is seen against reovirus, the RNA virus with cytoplasmic replication utilizing a viral RNA polymerase.

The target of the tilorone polymers is most likely a cellular RNA polymerase located in the nucleus associated with the production of viral and cellular mRNA, RNA polymerase II. This is supported by the finding that two viruses with their own virally-encoded RNA polymerases functioning in the cytoplasm of infected cells are mostly resistant to inhibition by both tilorone polymers.

Many cancers develop following mutations that accumulate in the cellular DNA replication machinery. A number of these modifications are also associated with viral infections as the invading viruses attempt to modify the cellular environment to promote and support vigorous virus replication. A recent report by Reeves and others [75] found the antitumor drug Gleevec capable of inhibiting vaccinia virus and raises the possibility that such drugs may be given at the same time as the vaccinia vaccination to prevent vaccine complications. 

Tilorone antiviral activity is most likely due to a direct inhibition of viral DNA synthesis for both HSV-1 and VZV-infected cells [75,76,77,78]. Tilorone itself is mostly used as an antiviral agent as its hydrochloride. It is the first recognized synthetic, low-molecular-weight compound that is an orally active interferon inducer, and is also reported to have anti-neoplastic and anti-inflammatory actions [79,80,81,82]. Tilorone hydrochloride has been shown to inhibit HSV-1 and vaccinia at concentrations of 10 μg/mL [83,84,85]. The researchers noted that the adsorption of the virus was not affected by the drug, and the penetration of the deoxyribonucleic acid of the input virus into the cytoplasm and nuclei proceeded normally when tilorone hydrochloride was present. However, newly synthesized viral deoxyribonucleic acid was not detectable under these conditions and there was a remarkable decrease in the rate of viral polypeptide synthesis.

The researchers tested a number of ssRNA viruses and no antiviral activity was measured. By comparison, we do see activity against a dsRNA virus, reovirus. This is not unexpected because reovirus, like a number of RNA viruses, requires cellular DNA synthesis to take place prior and/or during viral replication [86]. Tilorone, by inhibiting cellular DNA synthesis, can and does inhibit the replication of a number of DNA and RNA viruses.

For added comparison, the results for acyclovir are included. Inhibition of HSV-1 by acyclovir requires a concentration level of about 0.08 μg/mL and 2 μg/mL for VZV. This compares to a concentration about 10 μg/mL for the tilorone-Pt polymers against both of these viruses. But, in comparing the MIC values for acyclovir for MRV-3DE (250 μg/mL) and Vaccinia (200 μg/mL) the two tilorone polymers have much lower MIC values (10–20 μg/mL for Reovirus ST3 and 8–17 μg/mL for Vaccinia. Thus, the two tilorone polymers are broader acting antiviral agents in comparison to acyclovir and show promise as additional antiviral agents in the treatment against viral infections.

Tetramisole is an antihelmentic which acts on the cyclic nucleotide phosphodiesterases. It actually consists as a combination of optical isomers, the most active one being levamisole. Levamisole was the first synthetic chemical that exhibited immunomodulatory properties. It appears to restore normal macrophase and T-lymphocyte functions.

**Figure 13:**
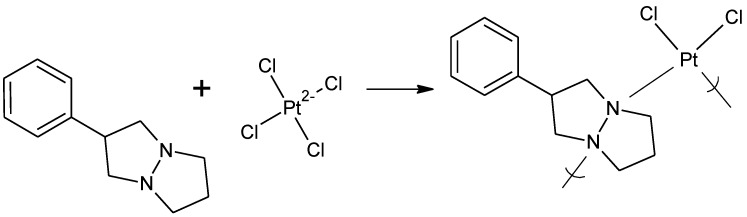
Repeat unit for the polymer obtained from reaction between tetramisole and tetrachloroplatinate.

Cisplatin polymer analogues, made through reaction of tetrachloroplatinate with tetramisole (Figure 13), were tested for their ability to inhibit EMC-D viruses that are responsible for the onset of juvenile diabetes symptoms in ICR Swiss male mice [87]. Other studies were undertaken showing that the polymer showed different activity profiles than the tetramisole, itself.

In a related study to that carried out employing tetramisole, a methotrexate polymer, was similarly tested. In the initial study female mice were treated. Generally, only male IRC Swiss mice develop diabetes-like symptoms. Female mice must first be treated with testosterone before they can develop diabetes-like symptoms. The mice were divided into three groups all of which received injections of the polymer (0.5 cc IP of polymer solution containing 6.4 × 10^3^ μg/kg). A week later groups I and II were treated with testosterone. On day eight, group 1 was again given a second 0.5 cc IP inoculation of the polymer solution. On day nine, all groups received 1 × 10^4^ pfu (plaque forming units) of EMC-D virus. On day 17, all mice were given a one hour glucose tolerance test. The glucose level for groups I and III were similar and significantly below the level for the diabetic mice in group II. This is consistent with the polymer effectively blocking the diabetogenic effects of the virus. Further, other results from this study were consistent with this strain of female mice being susceptible to developing diabetes-like symptoms even without the testosterone treatment.

A similar study was carried out except using male mice. Here, again, the polymer showed a greater positive effect on the control of diabetes than either of the reactants themselves. The glucose levels were near those of non-infected mice for the polymer-treated mice. Again, the incorporation of both the platinum and methotrexate into a polymer is the effective agent and not either of the drugs themselves. These two experiments are related to generation of a vaccine that can be employed to prevent onset of beta-cell damage by RNA viruses.

The third experiment focused on treatment subsequent to viral infection. The polymer was 100% more effective in viral control with delivery of the polymer one day after the mice were infected.

In summary, the methotrexate polymer is an effective antiviral agent against at least the EMC RNA virus.

Recently, we looked at the ability of methotrexate, tetrachloroplatinate, a physical mixture of methotrexate and tetrachloroplatinate and a methotrexate-platinum polymer to inhibit various viruses. In summary, methotrexate, the mixture, and the polymer are all active against HSV-1 and VZV viruses while the tetrachloroplatinate showed little or no activity against any of the tested viruses. The best inhibition, that is inhibition at the lowest concentration, was found for the polymer and the mixture consistent with there being some cooperative effect of the methotrexate and platinum moieties. It is possible that the mixture of tetrachloroplatinate and methotrexate may have formed polymer when mixed together in solution consistent with the similar findings found for the mixture and polymer.

In summary, the polymeric derivatives of cisplatin offer outstanding antiviral activity and deserve further work towards developing possible drugs for combating viruses.

## 3. Experimental Section

### 3.1. Synthesis of Bioactive Materials

The bioactive materials described in this presentation have been synthesized employing two well known and simple systems. The organotin polymers were synthesized employing the interfacial polycondensation system. Interfacial reactions are carried out under essentially non-equilibrium conditions and as such are not as sensitive to non-equal molar reactant amounts in achieving high polymer. The technique is herterophasic, with two fast-reacting reactants dissolved in a pair of immiscible liquids, one of which is usually water. The aqueous phase typically contains the Lewis base such as diol, diamine, or dithiol. The organic phase contains the Lewis acid, generally an acid halide such as in this case the organotin dihalides, dissolved in a suitable organic solvent such as hexane. Reaction occurs near the interface, hence the name. The interfacial synthetic process is employed industrially to synthesize polycarbonates and aromatic amines (aramids). For the present systems, reactions are rapid occurring generally within less than 30 seconds.

The platinum-containing polymers are synthesized employing a simple aqueous solution system. Briefly, each of the reactants, here the potassium tetrachloroplatinate and diamine, are dissolved in separate aqueous solutions. These solutions are mixed together with mild stirring. Polymer is produced as a precipitate after several hours.

In all cases, the reactants are available commercially. Thus, the polymers discussed in this review can be easily and rapidly synthesized employing commercially available reactants and commercially employed reaction systems.

**Figure 14:**
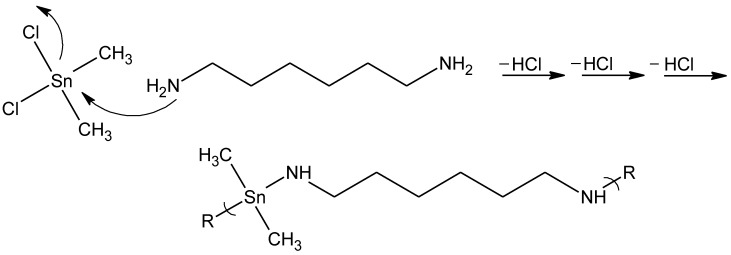
General reaction scheme for the reaction between dimethyltin dichloride and 1.6-hexamine to form a dimethyltin polyamine.

The two polymer types are synthesized employing two different approaches. For the synthesis of the organotin polymers, the products are produced employing a condensation mechanism where the tin on the organotin dihalide acts as an electrophillic site being attacked by Lewis bases such as diols and diamines, emitting HX for each reaction step (Figure 14).

**Figure 15:**
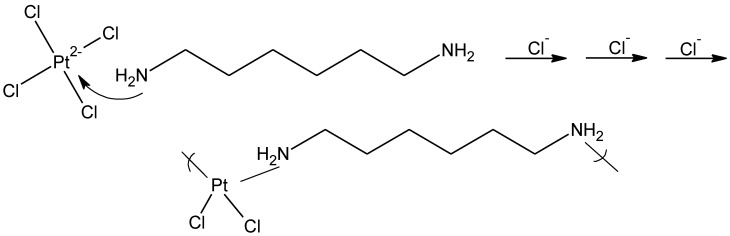
General reaction scheme for the reaction between tetrachloroplatinate and 1,6-hexanediamine forming the platinum-containing polymer.

In the case of the platinum derivatives based on cisplatin, the reaction is a coordination reaction where the diamine simply coordinates with the platinum (Figure 15).

On a stability level, under room conditions, the organotin polymers are generally stable for months to years in solution whereas the platinum-containing polymers are generally stable for weeks to months. Both are stable for years as solids. For our studies, the samples are maintained well below zero except when they are in the process of being tested.

### 3.2. Vaccinia and HSV-1 Antiviral Activity Evaluation

Initially, the drugs were evaluated with HSV-1 and vaccinia viruses (two DNA viruses). The activity of the drugs was tested to see if they inhibit the lysis of the WI-38 cells by the viruses. The WI-38 cells were be infected with each virus at a multiplicity of infection (MOI) of less than 1 in MEM without serum and with the highest concentration of the polymeric drug tested that was not toxic to the WI-38 cells. WI-38 cells were be seeded in a 96 well plate at a concentration of 10,000 cells per well. After an overnight incubation, the media removed and 50 µL of the media containing the polymeric drug and virus added. The plate was incubated for one hour, and rocked every 15 minutes for the duration of the hour. After the one hour incubation, 150 µL of media containing the appropriate concentration of the polymeric drugs was added. The vaccinia infections were incubated for 10 hours, the HSV-1 infections for 96 h. After the incubation period, the CellTiter 96® AQ_ueous_ One Solution Cell Proliferation Assay was used to determine the amount of cell lysis in each well. The % viability of the polymeric drugs was compared to a no drug viral infection and a no drug, no virus control. All experiments were done in triplicate. The polymeric drugs that protect at least 30% of the cells when compared to a no drug viral infection were tested further.

### 3.3. Vaccinia and HSV-1 Plaque Assays

WI-38 cells were plated at 80% confluency in 24 well plates. After an overnight incubation, the media removed and 100 µL of the media without serum containing the polymeric drug and virus (MOI < 1) was added. After a one hour incubation, rocking every 15 min, 500 µL of MEM with 10% FBS and 1X APS was added to the wells. The vaccinia infections were incubated for 10 h, the HSV-1 infections for 96 h. The plates were sonicated to release the entire virus in the cells and the samples stored at −20 °C until the plaque assays performed. The infections were done in duplicate. For the plaque assays, the viral samples were serially diluted from 10^−1^ to 10^−4^ in MEM without serum on the day of the infection. 143 (vaccinia) and vero (HSV-1) cells were plated at 80% confluency in 12 wells plates. After an overnight incubation, the media was removed and 125 µL of the serial dilutions viral samples from the WI-38 infections added. After a one hour incubation, rocking every 15 min, one milliliter of MEM with 5% BCS was added to each well. After a 48 h incubation, the media was removed, and crystal violet added to the cells. The viable cells were stained with the crystal violet, allowing the plaques to be counted. The wells that contain between 30 and 300 plaques were counted and the viral titer calculated. The GI_50_ of the drugs with the WI-38 cells and the concentration of the drug that inhibits 50% of the virus were compared. The plaque assays were done in duplicate.

## 4. Conclusions

This review of current studies examining the antiviral activity of metal-containing polymers represents only a beginning in the quest for urgently needed antiviral agents. We have focused on organotin and cisplatin-like polymers, an area with the most activity. The initial results indicate that further study is merited and that these two groups of metal-containing polymers may indeed represent two families of potential commercial antiviral drugs. This paper is intended to alert others of the potential use of metal-containing polymers in the war against viruses.

In general there needs to be a better correlation between the particular viruses inhibited and the structures of the compounds responsible for this inhibition. Further, after appropriate screening the compounds need to undergo *in vivo* testing. As this is occurring, mechanistic and site(s) of activity need to be investigated. Towards these ends the following need to be accomplished.

A number of studies will need to be carried out to demonstrate the ability of metal-containing biologically active materials to combat particular viruses. Along with continued screening, additional viruses should be studied. Such studies will not only identify additional candidates, but they may assist in identifying the site and mode of activity.

From the current studies with organotins and Group IVB metallocenes, the products from the Lewis bases hydroquinone and diethylstilbestrol exhibit the best promise as antiviral materials. In fact, with the exception of the organotin polyethers derived from ethylene glycol and PEG, all of the products that exhibit an ability to inhibit viral growth are derived from Lewis bases that contain pi-bonding that offer the ability to pi-bond with elements within the cell or viral organism to interact with and to effect their inhibition and/or destruction. By themselves, these Lewis bases exhibit little or no antiviral activity but combined with the organotin moiety they exhibit a range of activities. These products, along with the ethylene glycol and water soluble PEG products, merit further testing against other viruses and *in vivo* evaluation against the particular viruses where they exhibit good inhibition.

Along with the materials that have already shown promise, other compounds that exhibit good activity against particular viruses should be tested *in vivo* to determine if they continue to show antiviral activity.

The future for these compounds rests on the ability of the chemists and biochemists to convince the biologists and virologists, and more importantly the funding agencies, that organometallic polymers are viable as drugs to treat human infections. Laboratory and cell culture data continues to accumulate, or in some cases remain buried in the literature, but little work has been done to define the toxicity and possible efficacy of these compounds in humans.

In the next 10 years, in preparation for phase I clinical trials in humans, we expect/hope to see safety pharmacology studies conducted in rats and extended to dogs. Pharmacokinetic and metabolism studies, as well as general toxicity studies, need to be also undertaken in rats followed by reproduction toxicity studies. A strong record of compound discovery and cell testing exist but to move these compounds forward the absorption, distribution, metabolic and excretion profiles of these compounds in animals, must be produced. Further, incorporation of dyes such as fluorescein into these polymer backbones can be easily accomplished allowing for additional identification of specific sites and organs where these polymers concentrate.

These potential antiviral drugs are all synthesized rapidly from commercially available reactants employing techniques that are already industrially utilized to synthesize millions of tons of materials. Thus, the ready availability of these agents is apparent.

There exists a correlation between ability to inhibit cancer cell growth and ability to inhibit viral replication. Further, compounds derived from known antibacterial agents show the ability to inhibit bacteria, cancer cell lines, and viral replication contributing to the ongoing increase in data that demonstrates the common cellular pathways utilized during tumor growth and bacterial/viral infections [88,89,90,91,92,93,94].

In summary, organotin polymers exhibit viral reduction specific to the nature of the organotin and Lewis base nucleophiles. With the exception of the organotin polyethers derived from ethylene glycol and PEG, the first water soluble organotin polymer, the Lewis base contains a site of unsaturation allowing the polymers to interact with various biological agents that may interfere with viral replication.

The cisplatin polymers inhibit viral replication at low concentrations and the tilorone polymers demonstrate viral inhibition at lower concentrations and with a greater variety of virus than the known antiviral drug acyclovir.
